# An Optimized Hidden Node Detection Paradigm for Improving the Coverage and Network Efficiency in Wireless Multimedia Sensor Networks

**DOI:** 10.3390/s16091438

**Published:** 2016-09-07

**Authors:** Adwan Alanazi, Khaled Elleithy

**Affiliations:** Computer Science and Engineering Department, University of Bridgeport, 126 Park Ave, Bridgeport, CT 06604, USA; elleithy@bridgeport.edu

**Keywords:** wireless multimedia sensor networks (WMSNs), optimized occlusion-free viewpoint and multimedia coverage, mobility, coverage, energy efficient hidden node detection

## Abstract

Successful transmission of online multimedia streams in wireless multimedia sensor networks (WMSNs) is a big challenge due to their limited bandwidth and power resources. The existing WSN protocols are not completely appropriate for multimedia communication. The effectiveness of WMSNs varies, and it depends on the correct location of its sensor nodes in the field. Thus, maximizing the multimedia coverage is the most important issue in the delivery of multimedia contents. The nodes in WMSNs are either static or mobile. Thus, the node connections change continuously due to the mobility in wireless multimedia communication that causes an additional energy consumption, and synchronization loss between neighboring nodes. In this paper, we introduce an Optimized Hidden Node Detection (OHND) paradigm. The OHND consists of three phases: hidden node detection, message exchange, and location detection. These three phases aim to maximize the multimedia node coverage, and improve energy efficiency, hidden node detection capacity, and packet delivery ratio. OHND helps multimedia sensor nodes to compute the directional coverage. Furthermore, an OHND is used to maintain a continuous node– continuous neighbor discovery process in order to handle the mobility of the nodes. We implement our proposed algorithms by using a network simulator (NS2). The simulation results demonstrate that nodes are capable of maintaining direct coverage and detecting hidden nodes in order to maximize coverage and multimedia node mobility. To evaluate the performance of our proposed algorithms, we compared our results with other known approaches.

## 1. Introduction

WMSNs are capable of capturing audio-video information by using low-cost cameras embedded with sensor nodes. The multimedia sensors provide substantial information related to a particular area of interest [1,2,3,4,5,6]. However, multimedia applications experience problems due to online media transmission challenges [7]. Several sources of energy waste include idle listening, overhearing, packet loss due to collisions, and packet overhead in the multimedia sense. One of the major sources of energy waste are the packet collisions that happen when two nodes try to transmit the packets simultaneously. As a result, this causes a partial or complete packet loss at the recipient node. The lost packets need to be discarded or retransmitted, which could be the source of the excess energy consumption waste and Quality of Service (QoS) degradation. To enable the on-demand multimedia services, we need to focus on multimedia-supported algorithms in WMSNs to determine the hidden node problems and compute the directional coverage. The existing IEEE 802.15.4 standard is based on the blind back off carrier sense multiple approach with collision avoidance (CSMA/CA), where the nodes check the channel before sending the data frame. If, the channel is free, then the node initiates the transmission process; otherwise, it reattempts after a certain time. However, this approach is only suitable when the nodes hear each other, and that could be a rare case in WMSNs [6]. In most cases, the network coverage is much larger than the single node’s coverage area. In general, a well-defined coverage area does not support WMSNs because the propagation characteristics are uncertain and dynamic. In this situation, the node is unable to determine the receiver side. The results could be the probability of hidden node collisions. The restrictions of the multimedia sensing proficiencies relate to the location coverage and hidden node problem. Once a better location coverage and hidden node solution for multimedia sensors are discovered, the results will help to improve the capabilities of WMSNs applications. Additionally, WMSNs restrictions are caused by tall buildings, mountains, and trees. Hence, directional coverage of multimedia sensors could be completed once they are deployed in an area of interest. However, proper directional location for multimedia sensors requires correct field information prior to deployment of sensors. It is also likely that multimedia sensors might change their location due to mobility over time. This problem can be resolved by dynamic updates of the locations through location information exchanges [8]. However, multimedia applications have limitations that will affect the successful media transmission in the sensor networks. Node connectivity is subject to change because of wireless commotions [9,10]. When a sensor is responsive to its immediate neighbors, it must uninterruptedly upload information about its surroundings. 

The connectivity is a severe problem subject to the mobility change when the network has been set up. The sensors nodes try to identify new neighbors to address mobility problems, but a hidden node problem is a hurdle. Initial neighbor node discovery is typically performed when the sensor node has no proof about the configuration of its immediate neighbors. In this situation, the sensor node is unable to communicate with either the base station or the sink station. Thus, immediate neighboring nodes should be detected as soon as possible to set a path to the base station and contribute to the operation of the network [11,12]. Hence, in this state, more wide-ranging energy use is justified.

In order to handle the hidden node and coverage problem, our approach contributes the Obstacle-driven Negative Effect Strategy (ONES) method that handles the negative effect of the obstacles. The proposed method is designed for those scenarios where the number of the relays are less than those of relays required for building steady links. In addition, it is particularly suitable for those multimedia sensor networks that suffer due to several disconnected subdivisions of the network that are experiencing the issue of obstacles among the subdivisions. The method is validated by applying several assumptions and definitions. This helps reduce energy consumption and maintaining the QoS.

Furthermore, our paradigm contributes the Optimized Hidden Node Paradigm that involves the hidden node detection, message exchange phase, and location detection. Our paradigm is different from existing hidden node approaches, as we focus on the multiple discoveries of nodes rather than a single discovery of node. The approach is particularly designed for a distributed network as most of the existing approaches follow the central-based network in the node discovery process, which is also expensive for location-updating. We focus on improving the QoS and extending the network life. Thus, a network is divided into different subdivisions and is controlled by a coordinator node, as the subdivision process helps multimedia sensor nodes cover the entire area efficiently. The network life extension is justified with the proper selection of a controlling node using metrics such as residual energy, data forwarding capacity of the node, distance of the node from the base station, and memory allocation. These metrics are assigned the specific weightage that provides enough chance for each node to be a coordinator and balance the network. Handling the problem of overlapped subdivisions in the network particularly when a new joining node attempts to be a part of either subdivision is a critical issue that has also been handled by a priority-based synchronization.

The random wake-up procedure is applied to reduce the option of repeating collision amongst the nodes in the same subdivision. The beauty of our random wake-up process that it provides an opportunity for each node to coordinate with its neighborhood nodes to avoid the collision and initiate a faster discovery process for the new joining hidden node. In addition, each node applies an active discovery process to detect its coordinator node, whereas in the existing approaches, the coordinator (head node) is responsible for detecting its nodes in its subdivision, which puts an extra burden on the coordinator node. However, our approach handles this issue by assigning responsibility to each node to detect its coordinator node in each subdivision. Unlike existing approaches, our paper also contributes the novel location detection procedure that helps detect the maximum view, node boundary and viewpoint of multimedia sensor node; existing approaches either apply a node boundary or node view to detect the location. The remainder of the paper is organized as follows: Section 2 presents the salient features of the most related work. Section 3 presents an obstacle-driven negative effect strategy method. Section 4 presents optimized occlusion-free viewpoint and an energy efficient hidden node detection algorithms. Section 5 discusses the simulation setup and experimental results. Finally, the conclusions of the entire approach are given in Section 6.

## 2. Related Work

In this section, related WMSN approaches are discussed. Previous works discuss maximizing the coverage area and detecting the hidden nodes in the fields of wireless sensor networking, ad-hoc networks, and robotics. However, little research has been done in wireless multimedia sensor networking. Some addresses an omnidirectional coverage problem in wireless sensor networks [13,14], but it is not suitable for a bidirectional and an occlusion-free viewpoint. Numerous applications require bi-directional coverage, but existing coverage models are only suitable for traditional wireless sensor networks (WSNs), and do not support WMSNs. An initial study regarding the coverage of multimedia sensors is described in [15]. In this work, the authors proposed a routing protocol with the field of view camera placed on the floor. The video sensors are used by oceanographers to monitor the shallows. Furthermore, triangular view segments are used for calculating the coverage of wireless multimedia sensor networks in [16]. 

The neighbor discovery node process is proposed in [17] to regulate the new nodes from the base station. This approach only focuses on finding hidden nodes and not on energy consumption. The base station starts the node discovery process by broadcasting a HELLO message, and the node initiates the registration process after receiving the HELLO message. The node can switch channels to find the best HELLO message, which helps to locate the hidden nodes. In order to reduce the neighbor node discovery time, [18] introduced the HELLO message-based approach to identify the hidden nodes, but energy efficiency was not considered. In [19], an energy efficient node discovery algorithm was introduced based on temporal patterns of coincidences in order to reach other nodes. However, all these approaches address the wireless sensor network issues rather than multimedia wireless sensor network issues. In [11], the authors proposed the use of Voronoi diagrams and Delaunay triangulation to detect the best and worst coverage area in WMSNs. Another approach based on deploying an additional sensor node is introduced in [20] to maximize the coverage area. In this proposed approach, a two stage process was used for detection of phase coverage boundaries and obstacles by applying the formula $\sqrt{2\times R}$ (where *R*: sensing radius of sensors). 

In [8], virtual centripetal force-based coverage-enhancing algorithm was proposed for WMSNs. In this work, the grid theory, centripetal force model with essential mass and overlapping idea of the sensors are discussed. This algorithm shuts off any idle multimedia sensory to maximize the network coverage. Furthermore, the network is extended by redistributing sensors and by applying centripetal force based on the circular motion. The authors of [21] introduced a secure neighbor discovery process and attempted to protect the wireless sensor networks from different types of threats. The approach comprises a scalable key-distribution protocol that protects the neighbor nodes in the presence of malicious nodes. This aims to improve the secure neighbor discovery to guard the attacks of hidden nodes. The static network is deployed for securing the one-hop neighbor discovery process. However, the work does not address energy efficiency. The Line of Sight Method (LOSM) [22] is introduced for the wireless personal area network based on visible light communication technology. It handles the issue of the hidden nodes in IEEE 802.15.7 and in particular focuses on QoS parameters such as end-to-end delay, message loss, and goodput. The idle-pattern-based approach is introduced and in which the idle patterns are sent by the network coordinator to perpetuate the communication with other network sensor nodes. However, the work did not focus on energy efficiency and multimedia contents. The Efficient Beam Scanning Algorithm for Hidden Node (EBSAHN) [23] is proposed for the wireless sensor networks. This approach is based on an efficient Intra cluster grouping scheme (IC-GS) that helps add a new node into the wireless sensor network. Furthermore, it avoids the hidden node collision avoidance. The Hidden Node Problem (HNP) [24] is introduced in the wireless sensor network. This approach aims to generate the hidden node relationship for all nodes and allot the hidden nodes into different clusters. In this approach, time for super-frame is divided into sub-period, and size of the sub-period depends on the number of the hidden nodes into the cluster. The approach is primary based on improving the QoS. The Clustering-Based Mechanism for Detecting the Hidden Nodes (CMDHN) [25] is proposed for resolving the hidden node problem in the wireless sensor networks in order to improve the network performance. Furthermore, delay, throughput, and energy consumption are major parameters focuses. All existing approaches attempted to determine the hidden nodes and coverage problems but did not properly focus on energy efficiency, scalability, QoS, multimedia-content support delivery, and accuracy in multimedia sensor nodes. The characteristics and limitations of existing approaches are highlighted in Table 1.

## 3. Obstacle-Driven Negative Effect Strategy Method

Here, we consider the wireless sensor network that is divided into several subdivisions and suffered because of disconnected subdivisions; it also experiences the issue of the obstacles among the subdivisions. In the network, each subdivision is controlled by the coordinator node. The coordinator node has a communication range $‘R_{c}’$ that is the maximum Euclidean distance reached by the node’s radio. Our network is based on the following assumptions.

**Figure 1:**
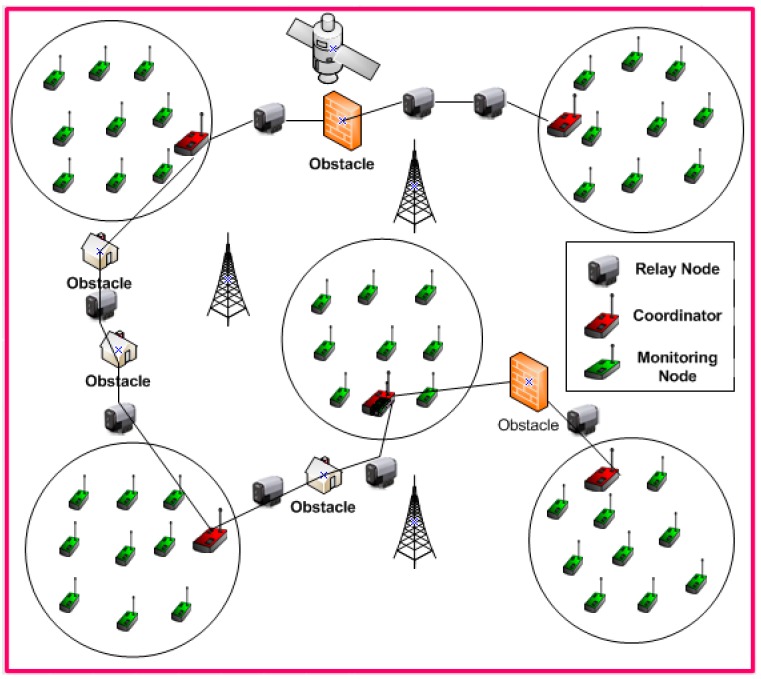
Showing the On-Demand Delivery process and obstacles.

**Assumption 1:**  All the relay nodes are mobile sensor and responsible for on-demand delivery as depicted in Figure 1.

**Assumption 2:**  There is at least one edge that interconnects the obstacles. Each obstacle is not overlapped with subdivisions.

**Assumption 3:**  *Let* ‘σ’ *be the count of subdivision* $‘Sd_{i}’$*. The number of available mobile relay nodes responsible for dealing with on-demand multimedia service *$‘M_{rn}’$ *should satisfy the following condition:*

$$M_{rn}>\sigma \;\&\&\;M_{rn}<T_{rn}$$

*where* $T_{rn}$*: Total relay nodes.*

**Definition 1:**  *Given an undirected graph* $‘G_{U}’$ *with subdivisions (segments)* ‘SD’ *and edges* ‘E’ *can be written as* $G_{U}=\left(SD,E\right)$*. Thus, the set of subdivisions are* $SD=\left\{Sd_{1},Sd_{2},Sd_{3},...,Sd_{n}\right\}$ *and edges.*

$E=\{(Sd_{i},Sd_{j})⎮Sd_{i},Sd_{j}\;\in \;SD\}$*. Let obstacle-driven negative effect strategy ‘Ψ’ be Ψ = (SD,* $E_{\Psi }$ *), where* $‘E_{\Psi }’$ *denotes the set of edges of (ONES). Let* $E_{d}=\left(E-E_{\Psi }\right),\forall \left(Sd_{i},Sd_{j}\right)\;\in \;E_{\Psi }$ *and* $ℾƸ(Sd_{p},Sd_{q})\;\in \;E_{d}\;˅\;r(Sd_{i},Sd_{j})<r(Sd_{p},Sd_{q})$*, where* $r(Sd_{p},Sd_{q})$*: Euclidean distance of subdivision* $Sd_{i}\;and\;Sd_{j}$*,* $E_{d}$*: deleted edges.*

**Definition 2:**  *The number of relay sensor nodes between subdivisions* $Sd_{i}\;and\;Sd_{j}$ *can be denoted by* $ℾƸ(Sd_{p},Sd_{q})\;\in \;E_{\Psi },\;M_{rn}(Sd_{i},Sd_{j}).$ *Thus,* $M_{rn}(Sd_{i},Sd_{j})$ *can be obtained by:*

(1) $$M_{rn}(Sd_{i},Sd_{j})=\lceil \frac{r(Sd_{p},Sd_{q})}{R_{c}}\rceil -1$$

**Definition 3:**  *Let* $T_{rn}$ *represents the total number of the relay needed to generate the steady network topology. Thus,* $T_{rn}$ *can be obtained by:*

(2) $$T_{rn}=\overset{N}{\underset{(Sd_{i},Sd_{j})\in E_{\Psi }}{\sum }}M_{rn}(Sd_{i},Sd_{j})$$

*Hence, obstacle-driven negative effect strategy can be simplified as* $SD=\left\{Sd_{1},Sd_{2},Sd_{3},...,Sd_{n}\right\}$ *that is the set of subdivisions and set of obstacles are* $O=\left\{O_{1},O_{2},O_{3},...,O_{n}\right\}$*. where* $O_{j}$ *cannot be overlapped with subdivision* $Sd_{i}$*. Thus,* $‘T_{rn}’$ *denotes the total relay nodes to create the steady network topology. Let* $M_{rn}=\left\{R_{a1},R_{a2},R_{a3},...,R_{an}\right\}$ *be the available relays for on-demand multimedia contents, where* $M_{rn}$ *satisfies following condition:*

$$M_{rn}>E_{d}\;⋀\;M_{rn}<T_{rn}$$

## 4. Optimized Hidden Node Detection Paradigm

An optimized hidden node detection paradigm is introduced for distributed wireless multimedia sensor networks because the sensor nodes are deployed in a disseminated manner within a realistic environment. On the other hand, centralized location deployment is not appropriate for WMSNs because these networks encompass a large number of multimedia nodes. Furthermore, updating the location is expensive within a centralized approach when compared to a distributed approach. Our approach consists of three phases:

- Hidden Node Detection
- Message Exchange Phase
- Location Detection

### 4.1. Hidden Node Detection

**Figure 2:**
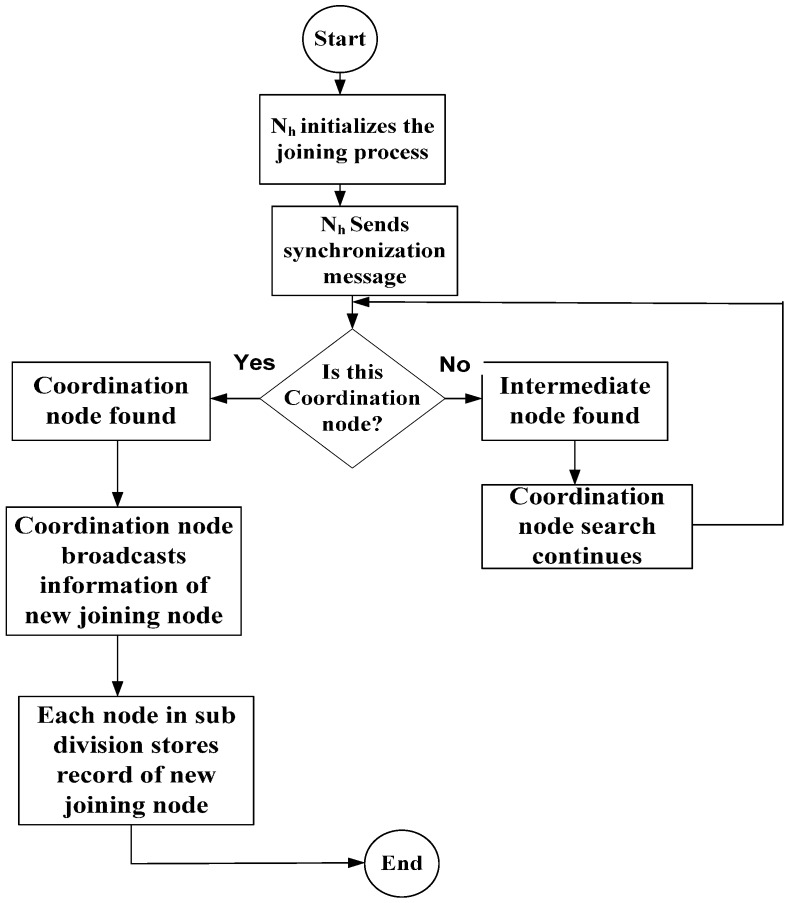
Coordination node synchronization process.

Detecting a hidden node in WMSNs is the critical problem that affects the network performance. Hence, the efficient neighbor discovery process helps in hidden node detection. In this phase, we focus on a continuous neighbor discovery process to determine the hidden nodes. Each sensor node uses a coordination-driven approach, and we chose the 1-hop multiple neighborhood discovery process rather than the particular node-discovery in the network that helps detect all hidden nodes at the1-hop neighborhood. As a result, the network consumes a minimum amount of energy and has a collision-free process. In this approach, the nodes share the schedule as discussed in [26]. The network is divided into different subdivisions, and each subdivision is controlled by a coordination node. However, our approach selects the coordination node based on the residual energy, data forwarding capacity of the node, distance of the node from the base station, and memory allocation. Each node continues to play a role as the coordinator until it possesses the higher weightage as compared with other nodes of the subdivision. The higher weightage is calculated by assigning different values as residual energy is assigned 33% weightage, the nearest distance of the node from the base station gets 25%, the data forwarding capability gets 15%, and the memory allocation resource gets 27% weightage. We tried to use different combinations of the weightage for each metrics, but we obtained the optimal results with our chosen weightage numbers. Thus, each coordination node is responsible for detecting the new hidden node when joining the network. Each new joining node is required to send a synchronization message to the coordination node, each subdivision has only one coordination node, which means there is less possibility of collision to handle synchronization message. In case a new joining node sends the coordination requests to two different subdivisions, if a node is located close to the subdivisions that are overlapped, the coordinator node that receives the first synchronization message that entertains the node. On other hand, the coordination node that receives a later synchronization message that responds to the new joining node, but that node has already become part of the other subdivision. As a result, the possibility exists for energy waste of the coordination node. However, the wasted energy of the coordination node is negligible. The coordination node replies to the new node and sends a message to all the nodes in the same subdivision to store records about the new joining node. After getting a message from the coordination node, all the nodes send a message to the new node. When it receives these messages, the new node sends a message to the coordination node about the nodes who replied to the previous message. The goal is to confirm the new node request and inform all the nodes in the same subdivision about the new member and the new node has the information about the other neighboring nodes. The synchronization message is dispersed over the entire links of the network to link with the coordination node. This is the way that the coordination node determines a new joining node is detected. When the coordination node has information about a new joining node that broadcasts within its subdivision nodes, the coordinating node will send out a message to all the nodes in the same subdivision. The synchronization process of a new joining node with the coordination node process is depicted in Figure 2.

The hidden node detection process applies a random wake-up procedure to reduce the option of repeating collisions amongst the nodes in the same subdivision. In this phase, each node coordinates with its neighborhood nodes during the wake-up period to avoid collisions and make a faster discovery process of any new joining hidden node. The wake up time period is very small, and the time of forwarding the HELLO message is even smaller. In this case, there is a possibility that two nodes can be active at same time and initiate the neighbor node discovery process. Therefore, we use a scheduling method to control the wake up process of two nodes at the same time. During the scheduling, the nodes are required to be synchronized with each other and report to the coordination node. During the scheduling, each receiving node chooses time slots and obtains the data during those time slots. The time slot process is performed without contradicting the schedule of the other node. This is the reason that the neighbor nodes are subdivided into different subdivisions, where each node chooses its slot assigned to that subdivision. Each sensor node decides randomly when to initiate the transmission of a HELLO message. If its message does not strike with another HELLO message, the node is referred to as a discovered node. We can also determine residual energy and the load of each node after the node discovery process occurs.

Let us assume that each sensor node communicates at the distance of the single-hop node to detect the hidden nodes. Each sensor node sends the HELLO message ‘Hm’ at the distance ‘d’ within the subdivision ‘Sd’ and is located at the N × N area of WMSN. The residual energy of the two types of multimedia sensor nodes, the coordinator node ‘C’ and the non-coordinator node ‘Cn’, can be determined as follows.

The coordinator node performs four types of jobs that include the synchronization with newly joined nodes, broadcasting the information of newly joined nodes inside the subdivision, scheduling the transmission of subdivision, and data collection from non-coordinator nodes of the subdivision. The synchronization process between the newly joined node and the coordinator is explained in Algorithm 1. 

**Algorithm 1**: Priority-based synchronization process between coordinator and newly joined nodesInitialization: ($C_{n1}$: coordinator node-1; $C_{n2}$: coordinator node-2; $N_{j}$: newly joined-node; $B_{m}$: beacon message for joining the subdivision; $S_{n}$: node synchronization process; $N_{sdi}$: subdivision of the network; ℓ: listening)Input: ($B_{m}$)Output: ($S_{n}$)Set $N_{j}$ attempts for $N_{sd}$ // New node intends to join the subdivision of the network$N_{j}$ ⏊ $B_{m}$ // New node sends beacon message for joining the network$B_{m}$ ℓ $C_{n1}$ && $C_{n2}$ // Beacon message sent by new node, but it is heard/listened by two coordinator nodes when two subdivisions are overlapped.$If\;C_{n1}\;\ell \;B_{m}\;\in \;N_{j}$ then // If coordinator node-1 gets beacon message from new node for joining the subdivision.$C_{n1}\;ɀ\;N_{j}\;\in \;N_{sd}$ // coordinator node-1 allows ‘ɀ’ the new node to be part of subdivision$C_{n2}Ґ$ $N_{j}$ // coordinator node-2 discards ‘Ґ’ the request initiated by new nodeElse if $C_{n2}\;ɀ\;N_{j}\;\in \;N_{sd}$ // If coordinator node-2 gets beacon message from new node for joining the subdivision$C_{n2}\;ɀ\;N_{j}\;\in \;N_{sd}$ // coordinator node-2 allows ‘ɀ’ the new node to be part of subdivision$C_{n1}\;Ґ$ $N_{j}$ // coordinator node-2 discards ‘Ґ’ the request initiated by new nodeEnd ifEnd else

Here, we determine the energy consumed for four types of jobs. Thus, the residual energy of ‘C’ and ‘Cn’ can be calculated as follows:

(3) $$C_{es}=\left[d^{2}\left\{\left(M_{s}\times \left(E_{r}\right))+(M_{s}\times \left(E_{a}\right)\right)\right\}\times S_{n}\right]$$

Equation (3) shows the consumed energy by coordinator node for synchronization:

(4) $$E_{b}=E_{r}+E_{a}$$

Equation (4) shows the consumed energy for broadcasting the message (disclosing the information of newly joined nodes to the subdivision nodes):

(5) $$E_{s}=\overset{k}{\underset{S_{n}=1}{\sum }}\left(E_{r}+E_{a}\right)\times d^{2}$$

Equation (5) shows the consumed energy by coordinator node for scheduling with subdivision nodes:

(6) $$E_{dc}={\left[d^{2}\left\{\left(M_{s}\times \left(E_{r}\right))+(M_{s}\times \left(E_{a}\right)\right)\right\}\times S_{n}\right]}^{2}$$

Equation (6) shows the consumed energy by coordinator node for collecting and forwarding the data.

We can determine the residual energy of the coordinator node and non-coordinator nodes based on the energy consumption for four tasks given by Equations (7) and (8), respectively:

(7) $$C_{re}=C_{ie}-\left(C_{es}+E_{b}+E_{s}+E_{dc}\right)$$

(8) $$NC_{re}=C_{ie}-\left(C_{es}+E_{b}+E_{dc}\right)$$

Determining the node’s load is significant for hidden node discovery. The load factor ${‘N}_{l}’$ requires the buffer capacity that is calculated d by using the Equation (9).

(9) $$N_{l}=\left[\frac{N_{hello}}{N_{bm}}\right]$$

Table 2 shows the details of the notations used and their respective explanations.

**Figure 3:**
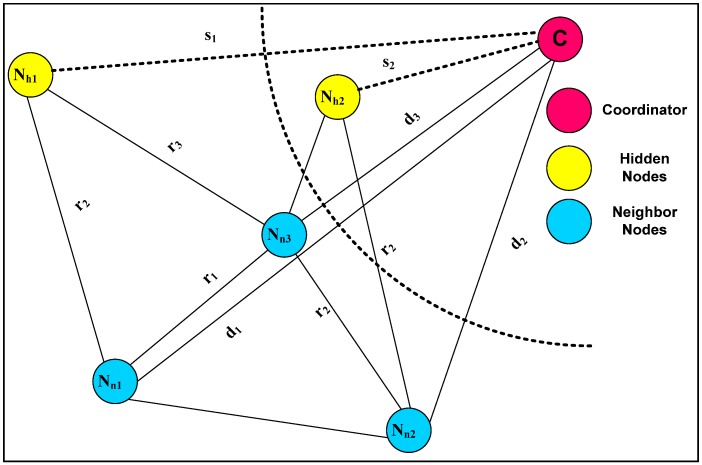
Hidden node discovery process.

When the nodes that are available at the distance of a one hop neighborhood node of coordinator node receive the messages they start calculating distance from the coordinator node to detect the hidden nodes depicted in Figure 3. Let us assume that coordinator node ‘C’ has three neighbor nodes: $‘N_{n1}’$, $‘N_{n2}’$ and $‘N_{n3}’$ with distance that calculates $‘d_{1}’$, $‘d_{2}’$ and $‘d_{3}’$ with known ranges $‘r_{1}’$, $‘r_{2}’$, $‘r_{3}’$ and $‘r_{4}’$ and $‘r_{n}’$ a hidden node $‘N_{h}’$.

Hence, the straight distance is at the possible values $‘s_{1}’$ and $‘s_{2}’$ from the coordinator node. The neighbor discovery method used in [27] is applied if there exist more than three multimedia sensor nodes. Thus, multimedia sensor nodes of more than three are connected either by $‘N_{n1}’$ and $‘N_{n2}’$ or $‘N_{n3}’$. We replace $‘N_{n1}’$, $‘N_{n2}’$, $‘N_{n3}’$ and $‘N_{nn}’$ with a new joined node or an existing hidden node that yields a pair of distance approximations. We can include more neighbor nodes to make a more accurate selection.

### 4.2. Message Exchange Phase

In this phase, the multimedia sensor nodes exist at a 1-hop neighborhood node that initiates the message exchange process to gather the information about the neighboring nodes. All sensor nodes use unicast addressing methodology that refers to the neighbor handshake indication (NHI) process. The NHI contains the identity of the nodes and the current location of the multimedia sensor node. Let us assume that the mobile multimedia sensor node comprises an identical standpoint and a list of overlapped neighbor nodes that are accessible in the same sensing position that yields the NHI. This aims to guarantee that each multimedia sensor node should detect their neighbor’s hidden nodes and their location:

(10) $$T_{n}=\overset{N}{\underset{i=0}{\sum }}(N_{ni}\;)$$

In Equation (10), the total neighbor nodes $‘T_{n}’$ are calculated to determine the exact number of all 1-hop neighbor nodes. Each neighbor node $‘N_{n}’$ sends on NHI message that can be obtained by:

(11) $$N_{n}=\overset{1}{\underset{j=1}{\int }}\left(N_{id}\right)+C_{l}$$

In Equation (11), multimedia sensor node unicasts the neighbor handshake indication process that contains node identity $‘N_{id}’$ and the current location $‘C_{l}’$:

(12) $$N_{n}\;\to R_{m}=\overset{1}{\underset{i=1}{\int }}\left(N_{id}\right)+C_{l}\times \overset{T_{n}}{\underset{N_{n}\;\in \;\beta }{\sum }}\left(\beta \right)\cdot N_{n}$$

In Equation (12), each neighbor node returns the message $‘R_{m}’$ with a node overlapping report ‘β’ including the current location of each neighbor node.

### 4.3. Location Detection

The location detection of the sensor node is of paramount significance for continuous communication. Thus, there are several events that can be implicitly adjusted and responded to only if the correct location of the event is detected. The location detection plays a key role for understanding the multimedia- application- contents. There are three advantages of detecting the location of the multimedia sensor node. First, location detection is required to determine the event of the interest. For example, the location of a fire, location of an intruder, or the location of the opponent’s tank in the arena are critical for deploying the relief troops and rescue squads. Second, location detection enables several application services, such as helping doctors to gain the information of medical gears and personnel in smart hospitals. Third, the location detection helps in several system functionalities, such as network coverage checking, geographical routing, and location-based information querying. This phase helps to detect a sensor’s maximum view. The location detection involves the node boundary and viewpoint. In the node boundary, we detect the area in which the node is capable of broadcasting the message. The viewpoint covers the degree of the node from 0 to 360 in which the node can be located at any degree. First, the node attempts to determine the location of the neighbor node within the boundary by broadcasting the message. If a node fails to locate the node within the boundary, then it initiates the neighbor distance search process by checking the distance of the node viewpoint of the neighbor node. Last, if the previous process fails, then an obstacle-distance process is used [4]. If the first two processes and later process fail, then the sensor node uses an optimized occlusion free viewpoint to determine the largest viewpoint [28]. Let us assume that sensor node $N_{s}$ is located in the node boundary area and has three obstacles inside the viewpoint that are relatively close to $N_{s}$. As such, these three obstacles restrict the sensor node from detecting the location of another node. Thus, we can obtain the location error rate by applying Equation (13):

(13) $$\theta \ell_{e}=\frac{\sqrt{{\left(l_{e1}-l_{a1}\right)}^{2}+{\left(l_{e2}-l_{a2}\right)}^{2}\times \Psi^{2}}}{T_{sn}}$$

The average location detection time is an important factor that helps determine the detection capacity of the multimedia sensor nodes. As such, this feature has an impact on the performance and extensibility of the network. Thus, Equation (14) provides an average location detection time for each multimedia sensor node as follows:

(14) $$\theta \ell_{t}=\overset{l}{\underset{i=0}{\sum }}\left(\frac{t_{ex}}{T_{sn}}\right)i$$

**Figure 4:**
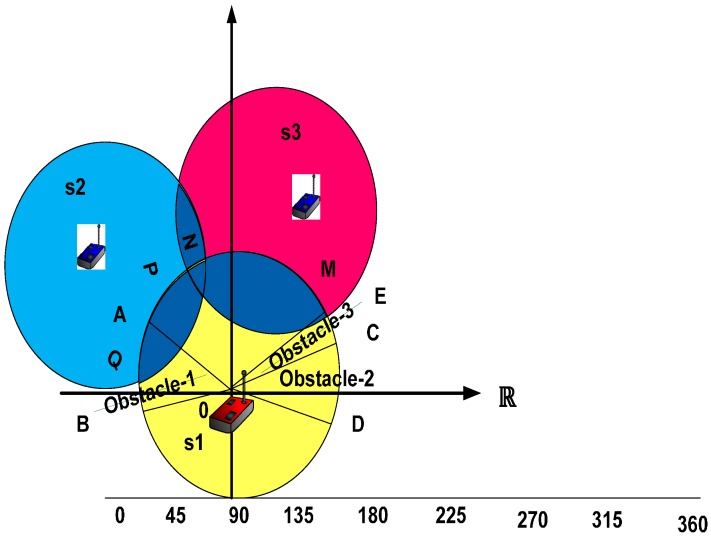
Obstacles within the node boundary and viewpoint; the yellow area represents the viewpoints of first sensor, the blue represents the viewpoints of the second sensor, the red represents the viewpoints of the third sensor and the dark blue represents the overlapped viewpoints area of the sensors.

In Figure 4, we show a complete node boundary process with the obstacles. The intersection of the curves on the given node boundary are displayed by including points A and B for the first obstacle, C and D for the second obstacle, and E and C for the third. Hence, the multimedia sensor node can determine whether there exists the obstacles Ψ that can be expressed as:

Ψ ∩<AℝB=0 , Ψ ∩<DℝC=0 & Ψ ∩<EℝC=0

then "Ψ" is visible to the viewpoint that refers to an AB clockwise curve; CD, and EC are available on the blocked curve of the viewpoint within the multimedia sensor node. This procedure not only finds the visible viewpoint, but it also helps detect the non-overlapped viewpoints. Similarly, the overlapped areas are detected by applying the node boundaries. 

Table 3 shows the details of the notations used and their respective explanations.

## 5. Simulation Setup and Experimental Results

To validate the effectiveness of our proposed optimized hidden node detection paradigm for wireless multimedia sensor networks, we performed the simulation by using network simulator-NS2. The network is constructed to cover 1200 × 1200 square meters. The 270 multimedia enabled nodes are randomly distributed with homogenous capabilities. The initial energy of each node is set at 7 joules. The simulation aims to identify the coverage and network efficiency in the presence of hidden nodes. Furthermore, the performance of OHND is compared with other known mechanisms handling the issue of hidden nodes: Line Of Sight Method (LOSM) [22], Efficient Beam Scanning Algorithm for Hidden Node (EBSAHN) [23], Hidden Node Problem (HNP) [24], and Clustering-Based Mechanism for Detecting the Hidden Nodes (CMDHN) [25].

We designed three scenarios that include both hidden nodes and without hidden nodes. In the first scenario, each multimedia sensor node hears 20 out of 30 of the other multimedia sensor nodes. This gives a 66.666% probability of detecting the hidden nodes without wasting additional energy. In the second scenario, 10 out of 30 multimedia sensor nodes can hear other nodes that give a 33.33% probability to detect the hidden nodes. In the third scenario, all end nodes that contribute in the network can listen to each other. The distance between each multimedia sensor node is set at 40 meters. These three scenarios demonstrate expected, worst, and ideal scenarios, respectively. All nodes are completely constructed using the angle $\theta =70^{{}^{\circ}}$ and the 30 meter sensing range that is set for each multimedia sensor node. The communication capability of each sensor node is set at 50 meters. We set 10–18 obstacles in the first scenario. In the second scenario, 18–27 obstacles were set. The third scenario had no obstacle. In scenarios 1 and 2, the viewpoint of the multimedia sensor nodes was affected. The remaining simulation parameters are given in Table 4.

Based on simulation, we obtained these results:

- Multimedia Throughput Efficiency
- Successful Packet Delivery Ratio
- Network Accuracy
- Energy Consumption with Hidden Nodes

### 5.1. Multimedia Throughput Efficiency

**Figure 5:**
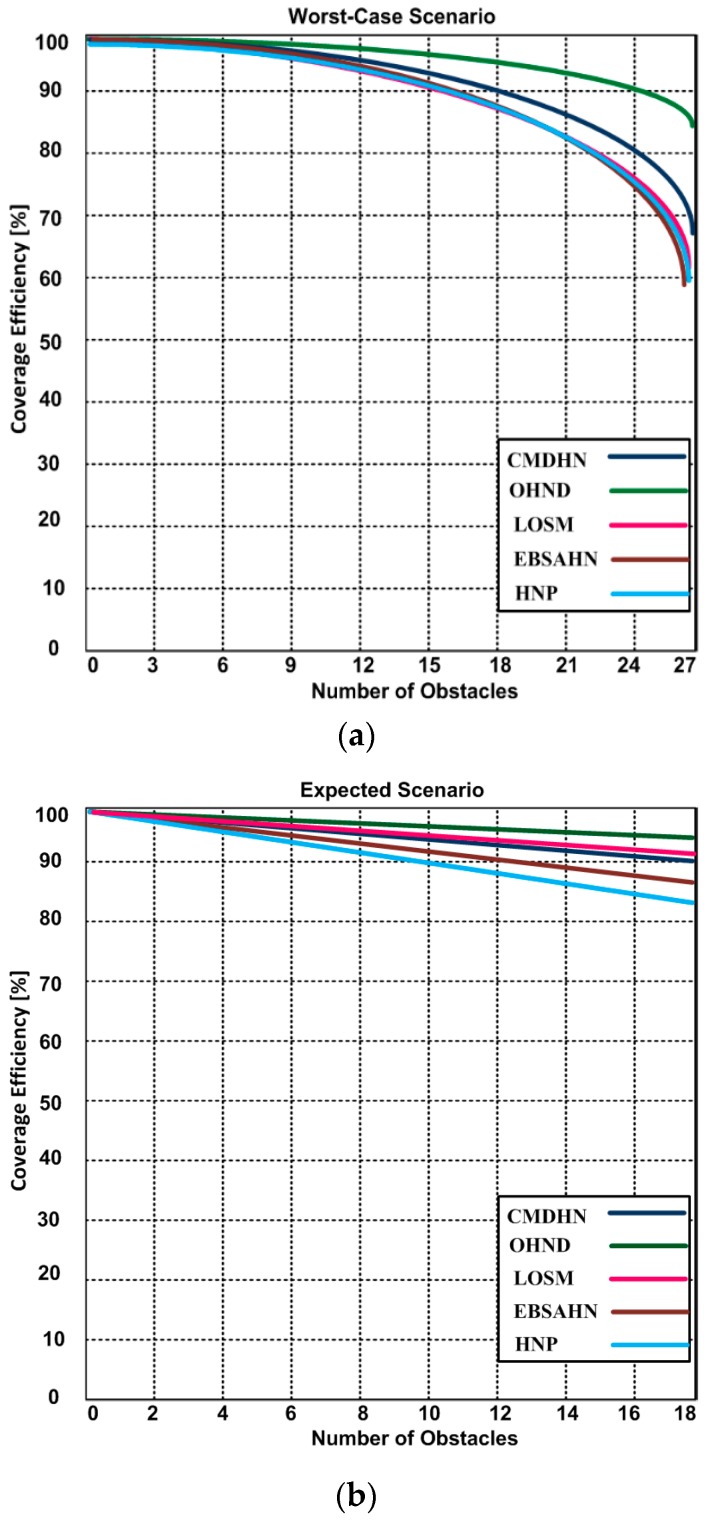

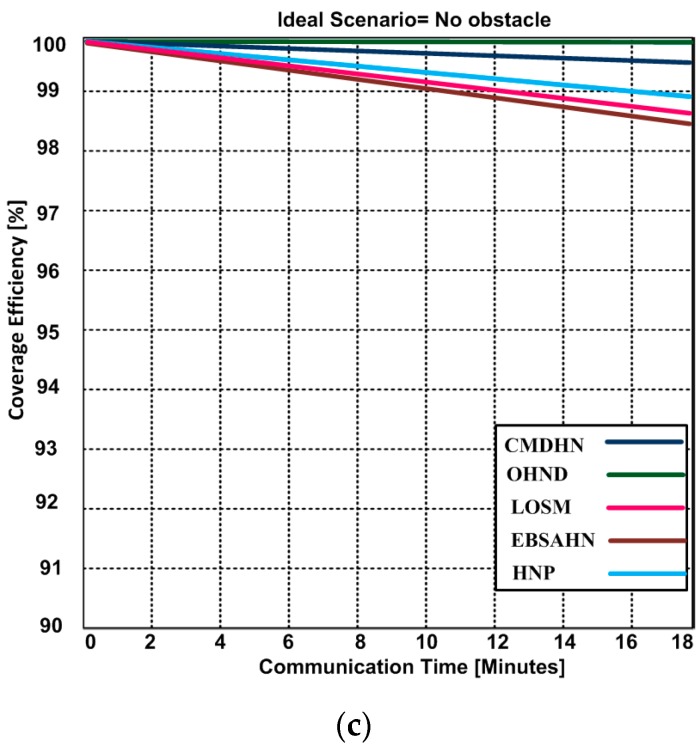
(**a**) multimedia converge in existence of 27 obstacles; (**b**) multimedia converge in existence of 18 obstacles; (**c**) multimedia coverage efficiency without the existence of obstacles.

To confirm the multimedia throughput-efficiency of our proposed optimized hidden node detection, we created three scenarios: the worst case, the expected case, and the ideal case. In the worst case scenario, we used 18–27 maximum obstacles depicted in Figure 5a. In the expected case, we used 10–18 obstacles as depicted in Figure 5b, and there were no obstacles in the ideal case depicted in Figure 5c. Furthermore, we compared the performance of OHND with LOSM, EBSAHN, HNP, and CMDHN. The communication time is set at 18 min in the three scenarios for all competing approaches. We observed in Figure 5a–c that when the number of obstacles increases, the throughput efficiency decreases. However, throughput efficiency of our proposed OHND is better than other competing approaches. Our results demonstrated that the approach of the OHND significantly improved the throughput efficiency of the multimedia sensor network. The reason for improvement in throughput efficiency, in our case, is the use of the obstacle-driven negative effect strategy method that handles the negative effect of the obstacles. Our approach is particularly suitable for multimedia sensor networks that suffer due to several disconnected subdivisions of the network that are experiencing obstacles among the subdivisions. Furthermore, the multiple discovery of nodes rather than single discovery of node substantially improved the performance.

### 5.2. Successful Packet Delivery Ratio

One of the drawbacks of the hidden node in WMSNs is to drop the packets due to collisions. As a result, the quality of service is highly affected. Successful packet delivery ratio can be shown in Equation (15):

(15) $$P_{s}=\frac{P_{d}\times 100}{P_{g}}$$

where $P_{s}$: Ratio of successful packets, $P_{g}$: Number of generated packets, and $P_{d}$: delivered packets.

**Figure 6:**
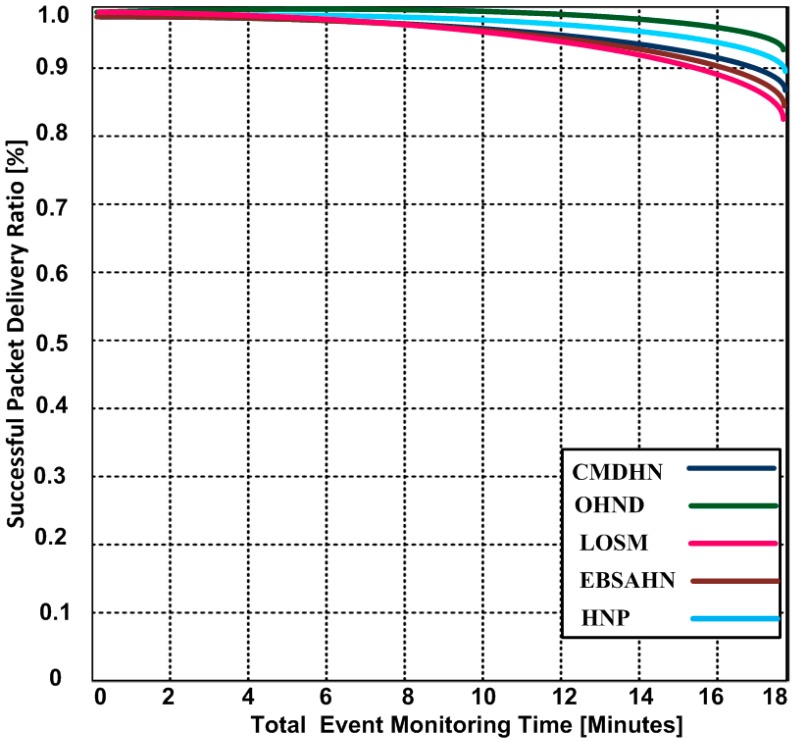
Successful packet delivery vs. number of monitoring events.

Figure 6 demonstrates the results of our proposed OHND and its comparison with LOSM, EBSAHN, HNP, and CMDHN approaches. The performance is measured by using a various number of events. When the number of events increase, the, successful packet delivery ratio starts reducing. However, our approach is more stable when compared with other competing approaches. Our approach has 93.4% of success rate after detecting 18 events, but other approaches have 82.24%–89.2% after monitoring the same number of events. In other approaches, the lost packets cannot be transmitted. In the presence of the hidden nodes, packets are lost. As a result, there is a possibility of collision that reduces the packet delivery ratio. 

### 5.3. Network Accuarasy

**Figure 7:**
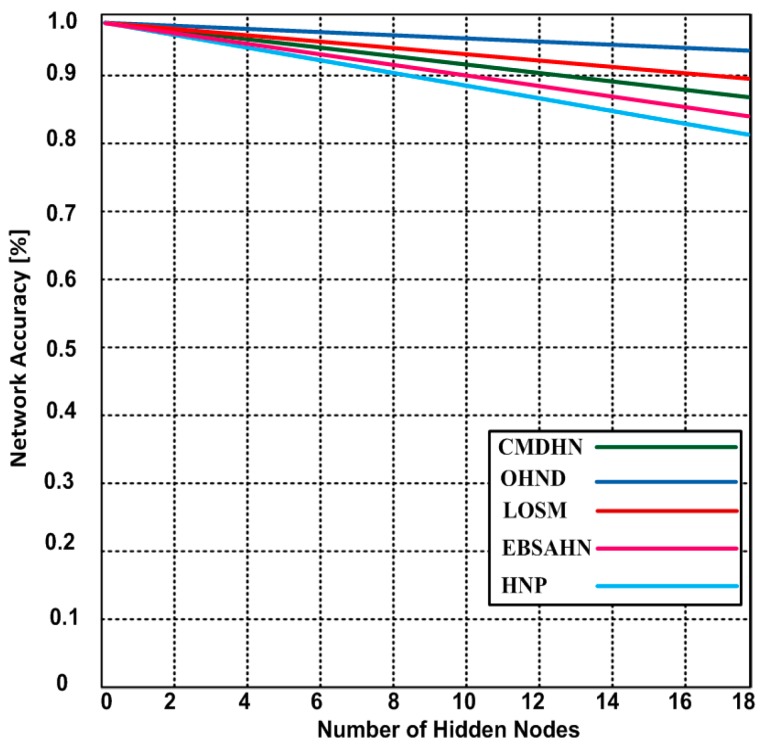
The network accuracy in presence of hidden nodes of OHND and other competing approaches.

Network accuracy is highly affected due to the presence of hidden nodes. Thus, end- to- end delay including processing, propagation delay, and time synchronization between the two end-to-end-points are extended. As a result, the network does not perform as expected and throughput performance is greatly degraded. In Figure 7, we determined the network accuracy of our proposed OHND and compared them with other competing approaches. When the number of hidden nodes increases, the network accuracy of our proposed approach is marginally reduced. On the other hand, the network accuracy of competing approaches is highly affected. In our case, our approach shows 96.1% network accuracy after detecting the 18 hidden nodes. HNP shows a lower network accuracy with an increased number of the hidden nodes. Furthermore, other approaches also showing lower network accuracy. The result demonstrates that our approach has an edge over other competing approaches.

The reason for better network accuracy in our case is the use of a random wake-up procedure that reduces the option of repeating collision amongst the nodes in the same subdivision. The beauty of our random wake-up process is to provide a chance for each node of coordination with its neighborhood nodes in avoiding a collision and initiating a faster discovery process for a new joining hidden node.

### 5.4. Energy Consumption with Hidden Node

**Figure 8:**
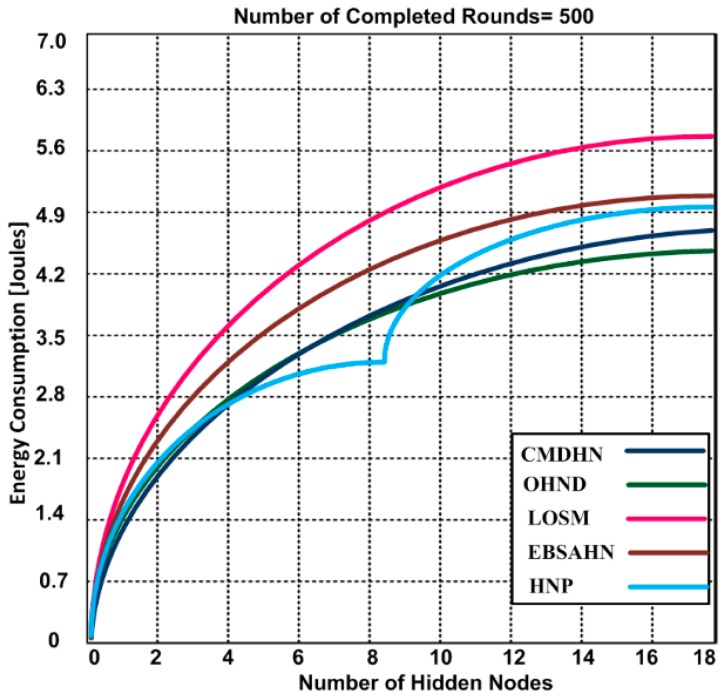
Energy consumption vs. number of hidden nodes.

The performance of the OHND approach was evaluated by using 500 rounds with a constant frame size of 512 data frames (including payload and data frame format). We performed several runs to determine the energy consumption of our proposed approach. Figure 8 demonstrates the energy consumption of our proposed approach and compares it with other competing approaches in the presence of hidden nodes using a maximum of 500 rounds. Based on the results, we observed that when the number of hidden nodes increase, the energy consumption rate also increases. However, our proposed OHND consumes less energy when compared to other competing approaches. HNP consumes less energy between 4–9 nodes, but when the number of hidden nodes increase, it performs poorly. OHND consumes an overall of 4.5 Joules with 18 hidden nodes using 500 rounds. However, other approaches consumed from 4.7–5.8 Joules for 500 rounds with a similar number of hidden nodes. The results demonstrate that our approach could also extend the network lifetime by saving more energy when compared to other approaches.

## 6. Conclusions

The hidden node problem creates a real threat to any type of multimedia wireless sensor network application. This paper introduces an optimized hidden node detection paradigm to improve the quality of service of the wireless multimedia sensor networks. Our paradigm consists of three phases: hidden node detection, message exchange phase, and location detection. These three phases resolve the hidden node problem and improve the network performance and QoS provision. The message exchange phase is responsible for detecting overlapped and non-overlapped areas of multimedia sensor nodes. The location detection phase determines the correct location of each multimedia node which helps improve the coverage efficiency of multimedia sensor nodes. Furthermore, the hidden node detection phase identifies the load and residual energy of the nodes when performing the discovery process. To determine the strength of our proposed OHND, we used NS2 and also compared the performance with other competing approaches: LOSM, EBSAHN, HNP, and CMDHN. The results demonstrate that our OHND improved multimedia coverage, energy consumption, and the packet delivery ratio when compared to other approaches. Furthermore, OHND has a hidden node detection capacity that is 0.8%–4.1% higher than the other the approaches. The simulation results confirm that our proposed paradigm is capable of determining hidden nodes for WMSNs application. In future research, we will implement our proposed OHND in a hardware-based environment.

## Figures and Tables

**Table 1:** The characteristics and limitations of the existing approaches.

Existing Protocols	Bio-Directional Coverage	Omni-Directional Coverage	Single-Directional Coverage	Energy-Efficient	Hidden Node Detection	Scalability	Multimedia Content-Support Delivery	QoS
Energy-efficient Probabilistic Area Coverage (EPAC) [12]		X		X				
Energy-efficient Node Scheduling Protocol for Target Coverage (ENSPTC) [13]		X		X				
Coverage Problem in Video-Based (CPV) [14]			X			X		
Optimal Worst-Case Coverage of Directional Field-of-View (OWCDF) [15]		X						X
Worst and Best-Case Coverage (WBC) [16]	X				X		X	
Delaunay Triangulation-Based Method (DTM) [17]			X		X			
Virtual Centripetal Force-based Coverage-Enhancing Algorithm (VCFEA) [18]				X				
Energy-Efficient Link Assessment (ELA) [19]			X	X				
Secure Neighbor Discovery (SND) [20]					X			
Line of Sight Method (LOSM) [22]					X			X
Efficient Beam Scanning Algorithm for Hidden Node (EBSAHN) [23]	X			X	X			
Hidden Node Problem (HNP) [24]			X		X			X
Clustering-Based Mechanism for Detecting the Hidden Nodes (CMDHN) [25]	X				X			X

**Table 2:** Notations used and their description.

Notation	Description
$C_{es}$	Consumed energy of coordinator node for synchronization
$M_{s}$	Number of synchronized messages by each newly joining node
$E_{r}$	Energy consumed by radio of multimedia sensor
$E_{a}$	Energy consumed for amplifying
$S_{n}$	Number of synchronizing nodes
d	Distance between coordinator and newly joined node
$E_{b}$	Energy consumed for broadcasting
$E_{s}$	Energy consumed by coordinator node for scheduling
*C*	Coordinator node
*C_n_*	Non-coordinator nodes
$C_{ie}$	Coordinator’s initial energy
$C_{re}$	Residual energy of coordinator
$E_{dc}$	Energy consumed by coordinator node for data collection
Sd	Subdivision
$N_{l}$	Node load
$N_{hello}$	Number of hello message by node
$N_{bm}$	Maximum buffer size of node
$N_{h}$	Hidden node

**Table 3:** Notations and their description.

Notations	Description
$\theta \ell_{e}$	Average location error
$l_{e1}$	First estimated location
$l_{e2}$	Second estimated location
$l_{a1}$	First actual location
$l_{a2}$	Second actual location
$T_{sn}$	Total number of multimedia sensor nodes
Ψ	Obstacles
$\theta \ell_{t}$	Average location detection time
l	Total locations of all multimedia sensor nodes
ℝ	Boundary range

**Table 4:** Simulation parameters and its corresponding values.

Parameters	Value
Size of network	1200 × 1200 square meters
Number of multimedia sensor nodes	270
Queue-capacity	50 packets
free-space propagation	47 meters
Maximum number of retransmissions allowed	03
Initial energy of node	7 Joules
MAC protocol	BN- MAC [6]
Size of packets	512 bytes
Data rate	450 kb/second
Sensing range of node	30 meters
Communication range	50 meters
Transmitter power	12.2 mW
Receiver power	13.4 mW
Buffer threshold	1024 Bytes
Sensing range	30 meters
Number of obstacles	10–18 for Expected and 18–27 for worst scenario
Simulation time	18 min
Average simulation run	10
Frame rate	40 frame/second
Reliability	[0.78, 0.92]
Average reporting rate	4 packet/second
Base station location	(0,700)
